# A qualitative analysis of interprofessional healthcare team members’ perceptions of patient barriers to healthcare engagement

**DOI:** 10.1186/s12913-016-1751-5

**Published:** 2016-09-20

**Authors:** Rhea E. Powell, Amanda Doty, Robin J. Casten, Barry W. Rovner, Kristin L. Rising

**Affiliations:** 1 Department of Medicine, Thomas Jefferson University, 833 Chestnut St, Suite 701, Philadelphia, PA 19107 USA; 2 College of Public Health, Temple University, Philadelphia, PA USA; 3 Department of Emergency Medicine, Thomas Jefferson University, 1025 Walnut Street, 300 Curtis Building, Philadelphia, PA 19107 USA; 4 Department of Psychiatry and Human Behavior, Thomas Jefferson University, 900 Walnut Street, 2nd floor, Philadelphia, PA 19107 USA; 5 Departments of Neurology and of Psychiatry and Human Behavior, Thomas Jefferson University, 900 Walnut Street, Suite 200, Philadelphia, PA 19107 USA

**Keywords:** Determinants of health/Population health, Healthcare needs and demand, Social determinants of health, Organization, Structure and delivery of healthcare

## Abstract

**Background:**

Healthcare systems increasingly engage interprofessional healthcare team members such as case managers, social workers, and community health workers to work directly with patients and improve population health. This study elicited perspectives of interprofessional healthcare team members regarding patient barriers to health and suggestions to address these barriers.

**Methods:**

This is a qualitative study employing focus groups and semi-structured interviews with 39 interprofessional healthcare team members in Philadelphia to elicit perceptions of patients’ needs and experiences with the health system, and suggestions for positioning health care systems to better serve patients. Themes were identified using a content analysis approach.

**Results:**

Three focus groups and 21 interviews were conducted with 26 hospital-based and 13 ambulatory-based participants. Three domains emerged to characterize barriers to care: social determinants, health system factors, and patient trust in the health system. Social determinants included insurance and financial shortcomings, mental health and substance abuse issues, housing and transportation-related limitations, and unpredictability associated with living in poverty. Suggestions for addressing these barriers included increased financial assistance from the health system, and building a workforce to address these determinants directly. Health care system factors included poor care coordination, inadequate communication of hospital discharge instructions, and difficulty navigating complex systems. Suggestions for addressing these barriers included enhanced communication between care sites, patient-centered scheduling, and improved patient education especially in discharge planning. Finally, factors related to patient trust of the health system emerged. Participants reported that patients are often intimidated by the health system, mistrusting of physicians, and fearful of receiving a serious diagnosis or prognosis. A suggestion for mitigating these issues was increased visibility of the health system within communities to foster trust and help providers gain a better understanding of unique community needs.

**Conclusion:**

This work explored interprofessional healthcare team members’ perceptions of patient barriers to healthcare engagement. Participants identified barriers related to social determinants of health, complex system organization, and patient mistrust of the health system. Participants offered concrete suggestions to address these barriers, with suggestions supporting current healthcare reform efforts that aim at addressing social determinants and improving health system coordination and adding new insight into how systems might work to improve patient and community trust.

**Electronic supplementary material:**

The online version of this article (doi:10.1186/s12913-016-1751-5) contains supplementary material, which is available to authorized users.

## Background

Health care systems are evolving to focus on providing more patient-centered, high-value care. In the United States, this shift has been operationalized through development of models such as the Patient Centered Medical Home (PCMH) and Accountable Care Organizations (ACOs). These models incorporate various interprofessional healthcare team members to help identify and intervene upon social, system, and patient-level factors that facilitate or impede care, with the end goal of improving population health outcomes [1, 2].

Interprofessional healthcare teams may include community health workers (CHWs), care managers (CMs), and social workers (SWs) who provide a range of community-based, practice-based, and hospital-based services, working directly with patients to navigate a complex system, better address health-related needs and reduce barriers to wellness. CHWs are community-based healthcare and public health workers who provide health education, navigation, social support, and outreach. Integrating CHWs into healthcare teams improves health behaviors [3], communicable disease management [4], and chronic disease outcomes, especially among vulnerable populations [5]. Similarly, CMs and SWs have demonstrated benefit on improving health-related outcomes. SWs have played a key role in patient-centered care for decades [6] and are integral to improving outcomes in primary care [7] and acute care settings [8]. CMs, often nurse-trained, are increasingly common on care teams and provide a variety of services including patient needs assessment, coordination of services and interventions, education, follow up, and patient advocacy [9]. These interprofessionals are being added to healthcare teams specifically because they have unique insights and skills in addressing patient needs. To date, however, work eliciting their perspectives has occurred primarily in specific disease settings, and largely outside the United States [10, 11].

In this work, we explored the perspective of interprofessional team members regarding the health-related needs of the patients and communities they serve, with the aim of applying these perspectives to inform the redesign of a more patient-responsive health system. Specifically, the goal of this study was to elicit the perspectives of community- and hospital-based inter-professional care team members regarding the challenges patients face in achieving wellness, and how the health system might better address these needs.

## Methods

### Study design and setting

This qualitative study enrolled interprofessional team members who provide patient care in Philadelphia, Pennsylvania. We engaged participants through focus groups and one-on-one interviews conducted from April 2014 to January 2015. A mix of focus groups and interviews was used to accommodate participants’ schedules and geographic convenience. As the research did not involve recruitment of patients or the review of medical records, the study was determined exempt by the institutional human research boards at both Thomas Jefferson University and The University of Pennsylvania.

### Selection and recruitment of participants

Adult English-speaking individuals (18 years and older) who are employed as interprofessional team members in Philadelphia were eligible to participate. Recruitment was led by a study team member with extensive experience working as a liaison between community groups and the medical system. She contacted potential participants by phone, email, and in person via networks developed throughout years of prior work throughout the city. In addition, emails were sent to CM and SW supervisors within the Philadelphia-based hospitals to be disseminated to all relevant hospital employees. Finally, flyers were posted in various city community organizations.

Focus groups were scheduled for a target size of 6–10 participants. Overall participant enrollment targeted a sample size of 40, with the final stop determined by thematic saturation [12]. Recruitment was structured to facilitate representative numbers of each of the targeted professions. Participants were given $20 for participation plus transportation costs.

### Data collection and processing

Focus groups and interviews were conducted by a trained facilitator with use of a moderator guide developed by the study team (Additional files 1 and 2). The same guide was used for both focus groups and interviews, with minor modifications as appropriate to make questions relevant for group versus individual discussion. Questions focused on identifying the types of assistance participants were asked to provide to patients, and the barriers and unmet medical and social needs that participants felt were most prominent for their patients.

Written informed consent was obtained by the session moderator. All focus groups and interviews were audio recorded. Participants completed a self-administered survey about basic demographics and professional experience at the end of each session.

### Analysis

Session recordings were sent to a professional transcription agency, and final transcripts were checked by members of the team for accuracy and to ensure de-identification of all information. Transcripts were imported into NVivo 10 [13] for analysis.

The codebook was developed using a content analysis approach. The goal of this approach is to classify interview data into distinct categories representing similar meanings without assigning pre-conceived themes to the interview data [14]. Members of the study team met to develop an initial coding framework with the initial two transcripts, which was refined with review of two more transcripts. The codebook was further refined through an iterative process with subsequent transcripts until consensus was reached. Coding was compared within and between transcripts to continue codebook refinement. The final codebook was reviewed by the entire team and was subsequently applied to the rest of the transcripts, and the initial transcripts were recoded with the final codebook. Double coding was performed on 25 % of the transcripts, with an average final kappa of 0.91.

After coding, comments were analyzed according to frequency by subject matter, category, and discipline of the respondent. We identified three primary categories (domains) that described the potential factors identified by participants that impact patients’ ability to manage their health: (1) social determinants of health—the economic, social, and political conditions of daily living that influence individual and community health [15, 16]; (2) health system factors—those factors related to the organizations and individuals whose role is to promote and restore health [17]; and (3) patient trust—the belief that healthcare providers and healthcare systems will provide reliable information and act in the best interest of their patients [18]. We describe findings, by domain, and include a final section that includes suggestions for improvement offered by study participants.

We use summary statistics to characterize the participants, and describe the major themes that arose within each of the identified domains.

## Results

We engaged 39 individuals in three focus groups and 21 interviews, with 26 hospital-based workers and 13 outpatient- and community- based workers. The mean age was 44 (range 22–64) and 87 % were female. Over 40 % of participants identified as social workers (Table 1).

**Table 1:** Participant Demographics

	N (%, 95 % CI)
Age in years – mean (range)	44 (22–64)
Female	34 (87, 72–95)
Education Level	
- High School/Associates Degree	10 (26, 14–42)
- College Degree	3 (8, 2–22)
- RN	7 (18, 9–34)
- Master’s Degree	18 (46, 31–62)
Profession	
- Community Health Worker	5 (13, 5–28)
- Social Worker	16 (41, 26–56)
- Case Manager	5 (13, 5–28)
- Clinical Resource Coordinator	4 (10, 4–25)
- Other^a^	9 (23, 12–39)
Work Setting	
- Hospital	26 (64, 47–78)
- Outpatient clinic	4 (10, 4–25)
- Community organization	9 (23, 12–40)

^a^Job titles included: Outreach coordinator, community empowerment manager, community leader, administration, nurse aide, outreach representative, health aide, and disease management coordinator

### Social determinants of health

Participants discussed multiple issues related to social determinants of health that limited their patients’ ability to manage their health including: insurance issues; financial barriers; mental health and substance abuse; and housing and transportation needs (Table 2).

**Table 2:** Social Determinants of Health

Theme	Quote
Uninsured: limited primary care availability and use of emergency department as primary source of care	*The folks in the community who use the health center. It’s so – it takes forever. You have to go there at 5:00 in the morning or whatever and stand in line and wait hours to get your meds and some people just don’t have time for that.* *They can’t go there and wait at 7:00 a.m. in the morning with the discharge instructions in hand half the morning to be seen to wonder if they’re gonna get a same day appointment or an appointment in a couple of days or so.* *If you don't have insurance, you go to the emergency room and you use the emergency room as your doctor, your clinic.* *Yes, and also they use the emergency room because I have found out through speaking with hundreds of people that they feel as though the emergency room have the services right there and they don’t have to wait like with a clinic you have to wait.*
Difficulty applying for insurance	*A fear that I have is that some people didn’t do it [enroll in ObamaCare], didn’t understand it, or even if they understood it didn’t do it the right way or what have you.*
Excessive copays	*I had a patient a few days ago who didn’t want a home visiting nurse to come out to check his incision because he couldn’t afford the co-pay.* *So what I’ve seen is there are very few options to a person without insurance and one of them being the health centers in Philadelphia… But often what I’ve seen with people without health insurance is also they can’t go to the health care center because it’s a sliding scale so it’s you pay to be seen and that doesn’t work for many people.*
Impact of mental health and substance abuse	*There’s no next, there’s no future… There’s no consequence all.*
Transportation logistics	*I find that logistics is the biggest issue with transportation… Thinking of new amputees living in [neighborhood] row homes, even getting up four steps is sometimes, it just can’t be done. And insurance companies don’t pay for a stretcher transport, so then you kind of have to coordinate with the entire family who can meet so-and-so at their front door and carry them up four steps to get in or out.*
Unpredictability	*I think most of us who are here today have a life that’s been somewhat organized and predictable and a lot of our individuals’ lives that we’re caring for has been anything but organized and predictable. And they are grateful to be awake and, you know, it hurts and you’ve no – you’ve no experience of what pain is manageable and what is not manageable*. *When you dig just a little deep and you open up the flood gates of what’s going on in their lives, its just mind boggling. So someone is perhaps not able to buy their insulin because they need to have 10 percent to get their son’s bail – to set the bail… I’m just saying that the problems are so pervasive from violence to depression to living in situations that are just – there isn’t any one service that’s gonna fix all this.* [*Primary care provider] implies stability. A [primary care provider] implies that the rest of your life has some degree of continuity. Sometimes you can’t – you can’t even control it.* *…so they feel that’s their life. It’s constant crisis and so that’s how they treat it. That’s why they go through the [emergency departments], the health centers… And I feel like even having continuity is so foreign in general because it’s never been in any pattern of their life in any area. So having one doctor follow you for 20 years—once in a while you get patients like that too, but—*

Lack of insurance was described as a significant barrier by many participants. Participants reported that despite availability of new health exchanges, patients often perceived insurance to be unaffordable, and many struggled to navigate the complex processes required to apply for and maintain insurance. Participants also explained that for patients without insurance, access to primary care was restricted due to excessive wait times and limited provider availability at city and community health centers: “*The folks in the community who use the health center. It’s so – it takes forever. You have to go there at 5:00 in the morning or whatever and stand in line and wait hours to get your meds and some people just don’t have time for that.”* Participants perceived that many of their uninsured patients often chose to go to the emergency department (ED) for care because of faster care and more available services.

Participants perceived that insured patients also faced financial barriers to health care. They described how the inability to pay outpatient and medication copays or meet high deductibles impacts use of care, resulting in many patients who were effectively under-insured: “*I had a patient a few days ago who didn’t want a home visiting nurse to come out to check his incision because he couldn’t afford the co-pay.*” These cost concerns led patients to delay care and to forego medications or other recommended treatments.

In addition to the impact of financial concerns, mental health and substance abuse issues were pervasive themes. Participants identified mental health and substance abuse issues as a root problem for many of their patients, and felt that patients lacked the resources to address these issues. These barriers were perceived to be a primary reason that many patients were unable to follow through with post-discharge plans or engage with regular outpatient services. Multiple participants felt unable to effectively motivate patients to pursue follow-up after hospital discharge in the setting of substance abuse or mental health struggles. As one participant explained: “*there’s no next, there’s no future… There’s no consequence all.”*

Participants described other socioeconomic issues that impacted patients’ abilities to regularly engage with primary care, including housing and transportation issues. Transportations issues included ability to afford transportation as well as logistical barriers such as mobility. As one community health worker explained, “*I find that logistics is the biggest issue with transportation… Thinking of new amputees living in [neighborhood] row homes, even getting up four steps is sometimes, it just can’t be done. And insurance companies don’t pay for a stretcher transport.*”

Finally, participants repeatedly brought up the impact of the unpredictable and complicated nature of living in poverty, especially as it impacted patients’ ability to prioritize and access primary care services. “*…so they feel that’s their life. It’s constant crisis and so that’s how they treat it. That’s why they go through the [emergency departments], the health centers… And I feel like even having continuity is so foreign in general because it’s never been in any pattern of their life in any area. So having one doctor follow you for 20 years - once in a while you get patients like that too, but…”*

### Health systems factors

At the health system level, participants talked about the “fractured” health system. (Table 3) They highlighted coordination problems at every step of the care process: primary care providers are unaware of hospitalizations, members of the inpatient treatment team give patients conflicting information, patients get discharged with incorrect medication orders, and information from one hospitalization is not communicated to the next care setting. These communication gaps were seen to be especially detrimental for patients transitioning home from the hospital: “*it seems like patients, are, they fall through the cracks.*” Patients lacked needed home services, had lapses in medications, and often didn’t receive needed follow-up care. Medication problems were seen as especially prevalent, with prescriptions given for medications that weren’t covered by a patient’s insurance, patients not being able to afford medication copays, and patients having medication toxicities that were not adequately assessed due to lack of follow up care.

**Table 3:** Health System Factors

Theme	Quote
Problems navigating the system	*I think the systems are completely broken and completely just un-navigatable… I have a Master’s degree, and I have trouble talking to the County Assistance Office. Think of the people that are actually using the County Assistance Office as their lifelines, if I can’t get through, if I have to wait days for a phone call back, what do you think those people are doing?* *I think there’s a misconception that if you have a primary care doctor that you're not gonna have any problems and everything’s gonna be fine and you can get an appointment tomorrow and that’s just not true. So whether you have insurance or you don’t have insurance there’s still – the systems are very hard to navigate…* *It seems like patients, are, they fall through the cracks.* *It’s not just I don’t have insurance, I’m not going anywhere; I have insurance and going somewhere. There’s people with insurance or Medicaid and then they go to get their insulin and their Medicaid got turned off for no reason without documentation, no phone call or mail to say that, and then they can’t get their insulin.*
Lack of understanding of value of primary care	*They do not understand the importance of having a primary care physician, what his role is, and how important it is to them maintaining your health and getting them the treatment that they need. So in their minds they can get this treatment without, they don’t know what they have one for, and so primary care physicians, going to the doctor’s, that kind of thing, it’s just something that is absent from them. And when they do take the time out to go to these doctors, it’s not good experiences, and I think because they’re not able to say what they mean and doctors don’t have the time to coach you through it.*
Emergency department as learned behavior	*Or they feel as though why waste my time going to the clinic when the clinic is going to send me – well the doctor at the clinic or my doctor is going to send me to the emergency room anyway, so I just don’t even bother with him and go straight there.* *I think patients rely too much on the emergency room for things. And some of that is just trained behavior. It’s, they, for example, patients that don’t get their medicine and they run out of it and they come to the hospital and we give it to them. So they learn that, oh, well, I’ll just go to the hospital and get it.* *If a patient calls and says, I’m short of breath, they (home care) say, go to the emergency room. Oh, we can’t send a nurse there to do emergency visits, which, they can’t. So those patients all fall through the cracks and they come in. The same with the primary care physicians’ offices. They’re overbooked, they’re very busy, the primary care doctors are in their offices, they’re in the hospitals, they’re running all around. When a patient calls with a bad problem, they say, go to the emergency room, we can’t see you here.*
Discharge instructions unclear	*Because when you’re being discharged from the hospital, it’s a blur of you talk to 10 different people about 10 different things and then you finally get home, and you’re like, wait, what? Why am I weighing myself?* *I think a lot of people say they understand because they don’t wanna come off as being – appearing stupid or intelligent, but they just also are overwhelmed by the experience and by whatever – maybe is being asked by them when they leave the hospital.*

Participants described how difficult it was for many of their patients to navigate a complex and fractured health system. “*I think the systems are completely broken and completely just un-navigatable…I have a Master’s degree, and I have trouble talking to the County Assistance Office. Think of the people that are actually using the County Assistance Office as their lifelines?”* For some patients, participants perceived an overall lack of understanding of the value of preventative and primary care. For others, participants discussed reliance on the ED for primary care as a learned behavior or strategy based on prior experience being sent to the ED after waiting in a clinic or calling for advice. “*Or they feel as though why waste my time going to the clinic when the clinic is going to send me [to the emergency room].”*

Discharge after inpatient hospitalization was brought up repeatedly during sessions as a particularly challenging time for patients, exacerbated by poor hospital care coordination. Participants explained that patients did not know what questions to ask, or did not feel comfortable asking questions at the time of discharge. They described patients being provided with insufficient information or with too much information without proper explanation. “*Because when you’re being discharged from the hospital, it’s a blur of you talk to 10 different people about 10 different things and then you finally get home, and you’re like, wait, what? Why am I weighing myself?”*

### Patient trust of the health system

Finally, participants spent extensive time discussing patient intimidation and general mistrust of the health system, as well as personal fears related to seeking and receiving diagnoses (Table 4).

**Table 4:** Patient Trust of the Health System

Theme	Quote
Doctors not interested in patients’ lives	*The doctor is just focusing on my medical needs and not focusing on my life issues or… looking at me as a human being.* *What doesn’t work is when you go in a room, when you meet a patient and you treat that patient as if that patient is a patient, which it is, but different than a social worker coming into the room.*
Lack of trust of primary care providers	*They don’t want to open up to them, because they’re afraid that they may call the Department of Human Services on them if the kids need milk and no Pampers.* *It seems like it has been passed on from maybe generations to generations the distrust of a doctor and saying, well, if I go to the doctor he is going to find – if I go for one thing, he’s going to find another thing or I can take care of myself and I don’t need to go to a doctor. It would just go away. So I find – I found that mistrust and then also thinking that you’ll be or I should say there’ll be judgment like someone is going to judge me. Somebody is not going to understand me, so I don’t want to deal with that, so they shy away.*
Fear of receiving bad diagnosis	*I think it’s fear. Fear causes people not to interact because you’re afraid of what you might hear. And then I found out with our family members, if you find out that cancer runs in your family all you have to do is have a spot and you’re already dying because everybody else has died.* *But because of fear you don’t ask something and a lot of people and people that we know die because they didn’t ask and they were afraid to what they may hear.*
Emergency department as safe haven	*And the [emergency department] is a safe haven, you know, that’s a place that you can go, get a meal, sit down on a bed for however long and just be safe and not have to worry about anything, because people are worrying about you at that point.* *It’s a fear of not knowing. A fear – I didn’t go to the doctor and I know this is it because most times people think them self into dying so I’m go to the emergency because I know I should have went earlier and I didn’t. So it’s like the last – that’s it.*

Participants explained that many patients felt intimidated by doctors and the health system, and that patients often believed that primary care providers were not motivated to understand their life situations, due to lack of time and lack of interest. “*The doctor is just focusing on my medical needs and not focusing on my life issues or… looking at me as a human being.”* This perception inhibited patients from discussing sensitive medical or social issues with their primary care providers, “*because they’re afraid that they may call the [Department of Human Services] on them if the kids need milk and no Pampers.*” As a result, patients did not form trusting relationships with their providers, thus they hesitated to ask questions, had limited follow-through with recommendations, and felt more comfortable seeking care in the emergency department.

In addition, participants indicated that fear of receiving a serious diagnosis prompts patients to delay care. “*Fear causes people not to interact because you’re afraid of what you might hear. And then I found out with our family members, if you find out that cancer runs in your family all you have to do is have a spot and you’re already dying because everybody else has died.”*

### Suggestions for improvement

Participants provided many concrete suggestions for how the health system could improve the care of vulnerable patients, touching on each of the domains outlined above (Table 5).

**Table 5:** Participant suggestions for improvement

Domain	Suggestions
Social Determinants	- Free medical care and services for some patients - Reduce financial barriers - Expand insurance programs to provide better access to primary care - Implement sliding scale copays - Financial incentives for provision of more care and social assistance to vulnerable populations - Expand capacity of inter-professional care team members to work flexibility with patients
Health System Factors	- Additional staff to help patients throughout transitions - Same individuals to help patients in hospital and at home (bridge the transition) - Patient-centered logistical coordination (i.e. follow-up appointments on same day) - Improved hospital communication from one visit to the next - Clear and understandable communication of discharge instructions - Better information sharing about community resources - Patient education about personal health management and the medical system
Patient Trust of the Health System	- Increase visibility of individual providers and health systems within communities - Establish partnerships between communities and health providers - Host community health fairs - Take more time to listen to patients to learn about their actual needs - Increase number of minority providers and staff - Include aspects of immigrant cultures into hospital environment - Shift towards more patient-centered care, instead of disease-centered approach

Suggestions for addressing financial issues included: providing free medical care and services to some individuals; expanding insurance programs to provide greater access to primary care providers; and implementing sliding scales for copays. In addition, participants suggested establishing stronger financial incentives for health team members to provide more extensive care and social assistance to vulnerable patients, and expanding the capacity for inter-professional team members to work flexibly to problem-solve with patients: “*I would like here, for us to have somebody, either myself or a nurse practitioner or someone that’s pretty well-trained, to be able to go to the patients who use the systems the most and really see what they’re doing in their houses and try to troubleshoot…Just go in and try to find the problems and solve the problems*.”

To improve care coordination across healthcare transitions, participants suggested having staff available to assist patients throughout the transition period as well as developing approaches to better patient-centered logistical coordination of transitional care. Both community- and hospital-based workers suggested that having the same individual in the hospital and at home to bridge “*the disconnect between the community and healthcare*” would help facilitate smooth transitions. Suggestions to improve logistical issues included scheduling multiple appointments in the same day, and insuring that patients had the means to adhere to treatment plans. “*We do not do a good job of trying to coordinate these services so these patients can come in and see the neurosurgeon, the ortho, the hand in one day. Come here for this. Come there for that… And then we turn around and we label them. You’re non-compliant. You’re this. You’re that. But we’re – we are more concerned with our own schedules and what makes life easy for us.”* Participants also suggested improved communication between and within hospitals from visit to visit, more clear and understandable communication of discharge instructions, and better information sharing around community resources.

Some participants thought that patients would benefit from general education on health and the healthcare system: “*I think there needs to be more education with families that don’t have any idea what the medical health system is. When you go out to places in [neighborhood] where there’s no clinics nearby. There’s no pharmacies nearby. There’s no people walking around on the streets that are talking about their healthcare.… I think people grow up in neighborhoods where they really don’t understand the value of going to the doctors.*”

Finally, there were numerous concrete suggestions for improving trust of the health system. Participants suggested that individual providers and health systems should have more visibility within the communities. They felt strongly that increased health system outreach to better identify community-specific needs and to establish partnerships between communities and health providers would strengthen community trust. Participants suggested targeted efforts by providers to be known in the community setting, for example by hosting health fairs. “*I always say take it to the streets because that’s where they are is in the street and to let them know that you care enough to walk the street to let them know this is what’s available to you lets them know that you care.”* In addition, they suggested that providers should take more time to listen to the patients to learn about their actual needs. “*I think doctors can become better listeners. I think people know their bodies…”* Other suggestions included: increasing the number of minority providers and staff in order to build community trust; including aspects of immigrant cultures into the hospital environment in areas with strong immigrant communities; and shifting towards more person-centered care as opposed to the current disease-centered approach used by most providers.

## Discussion

This study elicited the perspectives of interprofessional healthcare team members including SWs, CMs, and CHWs to understand challenges patients face managing their health and opportunities for the system to address those challenges. Study participants identified challenges related to social determinants of health, navigation of fragmented health systems, lack of care coordination, and mistrust of providers and health systems. There is a substantial body of literature surrounding issues study participants raised pertaining to the impact of social determinants on health [15, 19–21], and the need for effective health systems [22, 23]. Many of these issues are being targeted in current health reform efforts in the United States, including the establishment of accountable health communities [24], hospital community benefit activities [25], health information exchanges [26], telehealth programs [27], and population health models of care such as ACOs and PCMHs [28].

In contrast, to our knowledge, there are no national initiatives that have been designed specifically to impact patient trust despite multiple studies demonstrating that institutional mistrust (lack of trust that patients have in health systems) is associated with underutilization of health care [29], lower adherence to treatment plans [30, 31], and overall worse self-perceived health [32, 33]. A recent study suggests that mistrust may mediate disparities in utilization of primary care vs. the emergency department (ED) as a usual source of care [34], and our prior work identified fear both as a primary driver of ED visits [35] and a contributing factor for short-term ED return visits [36]. In our study, participants described patients’ lack of trust in primary care providers, and a sense that doctors are not interested in patients’ lives, as leading to lack of ongoing relationships with primary care providers and limited follow-through with treatment recommendations. Study participants additionally perceived patient fears – of being reported to regulatory agencies, of being misunderstood, of receiving a new diagnosis – as contributing to delays in seeking care and to limitations in following care recommendations. This study reinforces the existing literature on health system trust and mistrust, underscoring the importance of trust in the healthcare system to achieve favorable health outcomes, and expands the understanding of the impact of fear on healthcare utilization as a driver of both healthcare use and avoidance.

While the implications of trust and mistrust in healthcare providers have been established in a number of healthcare settings [29–33, 37–39], little is known about specific practices that build trust in providers and healthcare systems. Prior trust-building interventions studied have largely focused on training of providers, and have not demonstrated significant efficacy [40, 41]. Findings from this study add to the literature on trust and health by offering concrete suggestions for improving community trust in the health system. CHWs and other interprofessional team members are particularly attuned to issues of trust, as building trust and rapport with patients mediates their ability to improve patient health outcomes [42]. Suggestions to improve patient and community trust included increasing visibility and involvement of providers in the community by engaging communities directly in their neighborhood spaces. Specific examples included: providing health fairs in the communities they serve, introducing mobile clinics, and conducting health education activities with community partners. Other suggestions to build patient and community trust included employing a more diverse staff and educating healthcare providers on how to use a person-centered approach in caring for the communities they serve. These suggested interventions have not previously been tested as it pertains to trust between patients and health systems, and offer opportunities for future directions.

Our study has a number of limitations. The study did not capture any demographic information about the patients served by study participants, thus we are unable to comment specifically on this population. However, we recruited participants who work with patients solely within an urban city (Philadelphia) with a large underserved population. Thus, we anticipate that the population represented is disproportionally poor, underinsured, and has higher rates of non-medical problems that make navigating healthcare systems difficult. Although participants may have worked in other settings prior to their current jobs, the insights gained from this work may be specific to an urban underserved area, and may not be generalizable to other geographic regions. Additionally, participants were only able to speak to the needs of participants whom they had served, thus insights regarding individuals who had refused or were never offered services were not able to be included. It is possible that individuals who do not access the healthcare system or who decline referrals to the types of resources offered by these interprofessional team members may have different primary needs and perspectives than those captured within this work. Findings are also impacted by the make-up of the participants interviewed - hospital-based workers, for example, frequently highlighted issues related to patient discharge while community-based workers were more focused on outpatient issues. We included workers from both settings to ensure that we touched on issues relevant in both settings.

## Conclusion

This study offers important insights from a previously under-represented population within research, interprofessional healthcare team members, to understand barriers to achieving health in the communities, and provide concrete suggestions for how the health system can better address these barriers. Participants described social determinants, complex system organization, and patient fear and mistrust of the health system as the primary factors impacting healthcare engagement. Suggestions for improvement supported current healthcare reform efforts aimed at addressing social determinants of health and improving health system coordination, and added new knowledge regarding ways future efforts might specifically impact patient trust of the health system. Future work is needed to directly elicit patient perspectives on strategies to build trust in healthcare providers and systems and to test the impact of these strategies on measures of patient trust, healthcare utilization, and patient-centered outcomes.

## Additional files

Additional file 1: Understanding What Patient Needs to Stay Healthy: The Perspective of Community and Hospital-based Caregivers. Focus Group Guide for Community Health Workers (DOCX 29 kb) Additional file 2: Understanding What Patient Needs to Stay Healthy: The Perspective of Community and Hospital-based Caregivers. Focus Group Guide for Care Managers and Social Workers (DOCX 29 kb)

## Abbreviations

ACO: Accountable care organization
CHW: Community health workers
CM: Case manager
ED: Emergency department
PCMH: Patient centered medical home
SW: Social worker
