# Supplementary material for: A qualitative analysis of interprofessional healthcare team members’ perceptions of patient barriers to healthcare engagement
Source: BMC Health Serv Res. 2016 Sep 20;16:493. doi: 10.1186/s12913-016-1751-5 (PMC5028928; doi:10.1186/s12913-016-1751-5)
Supplement: Additional file 2: — Understanding What Patient Needs to Stay Healthy: The Perspective of Community and Hospital-based Caregivers. Focus Group Guide for Care Managers and Social Workers (DOCX 29 kb) [file 12913_2016_1751_MOESM2_ESM.docx]

**Understanding What Patients Needs to Stay Healthy: The Perspective of Community and Hospital-based Caregivers**

**Focus Group Guide – Case Managers and Social Workers**

**Introduction/Script**

Good morning/afternoon, and welcome to this discussion. I would like to thank everyone for taking the time to participate in this group meeting. My name is _____________ , my colleague is __________________and we work for the University of Pennsylvania. We are working to better understand what types of assistance and services patients need in their daily lives in helping them stay healthy. You have been invited to this group discussion because you are involved with providing care and services to patients to assist them after discharge from the hospital, which may include discharge from the emergency department, observation unit, or inpatient service, and thus are involved in identifying and potentially helping to fulfill needs that patients will have once they leave the hospital.

We are interested in your opinions and thoughts about things that patients most need help with in their daily lives, and what things they struggle with most in keeping themselves healthy. These may include things such as getting their medications, having enough food, contacting their doctors or getting clinic appointments when they have medical needs or questions, as well as a range of other things that you may identify. I want to make sure we respect the privacy of individuals, so I don’t want the details of any one specific individual, rather a general idea from you of the types of people you work with.

We are here to listen to your viewpoints, and there are no “right” or “wrong” answers. We want everyone to share freely, with one person speaking at a time as we are recording. I will make sure that everyone has a chance to participate in the conversation, because we are really interested in hearing different views. If there are questions you don’t wish to respond to, feel free not to.

Since your comments are very important, we want to make sure we have them all. We will audio record the session and then produce a transcript of all the comments. We will only use first names during the discussion. All of your comments will be treated with confidentiality. Your name will not be used or retained as part of the transcript. Are there any questions before we begin?

**The work you do**

First let’s get an idea of who you are and the type of work you do.

**Questions**

- ***Let’s go around the room for this first question - What is your name (just your first name), how long have you been working here in Philadelphia, and what is your role in caring for or providing assistance to patients?***
  - ***How do you describe to your friends what you do for your job?***
  - ***What hospital setting do you work in primarily? - ED, observation unit, inpatient***

**The ways you meet and interact with your clients**

I’d like to start with learning about how you initially meet your clients, and how you establish and maintain a connection with them.

**Questions**

- ***What do you do to gain your clients’ trust – what works and what doesn’t?***
- ***Tell me about times when you have continued to work with clients beyond what your job requires (additional duties, additional time)***
  - ***Do you communicate with your clients after they leave the hospital? If so, how? (email, phone, in-person visits, etc)***

**The patients you serve and their access to outpatient care**

Now I’d like to get a general understanding of the types of people you work with and their existing connections with outpatient doctors.

**Questions**

- ***Tell me about the challenges that your clients have in accessing outpatient care.***
- ***Do your clients usually have a primary care physician (PCP)?***
  - ***How are the PCPs usually involved in your clients’ care?***
  - ***How do your clients perceive the role of the PCP?***

**The primary needs of your clients**

*****DON’T HESITATE TO SKIP PARTS OF THIS SECTION**

Now let’s talk about the needs of your clients and the types of assistance that you provide to them.

**Questions**

- ***What are the most common needs (medical and non-medical) that your clients identify?***
- ***How do you fulfill your clients’ needs?***
  - ***What are the resources that you use most often to address their health needs. (clinics, health lines)***
- ***Tell me about the resources that your clients use most often to address their health needs.***
  - **The PEOPLE THEY CALL when they have health related questions or needs (including medical people, family or friends, etc)**
  - **The PLACES THEY GO most often to address their health needs, including clinics, emergency departments, health fairs, and other places.**

**The types of needs your clients have which are still most often unmet or under-addressed**

Finally, let’s talk about the needs of your clients which are most often hard to address or fulfill and how the social and medical systems as a whole could better address needs of the community.

**Questions**

- ***What are the needs that you find it hardest to provide assistance with?***
  - ***What do your clients ask for that you are not able to provide or fix?***
- ***What do you think the health system could do to make it easier for residents of the community to stay healthier and reduce their need to come to the hospital?***
- ***Similarly, what do you think the social system could do to better support the health of the community?***

**Final Thoughts**

Before we wrap up the discussion, I’d like to take a moment to ask two last questions. I’d like to just go around the table and ask each of you to respond to each question.

**1. What are the main reasons you hear from clients for why they decide to go to the hospital/emergency room?**

**2. What do you think the top 3 things are that the health system needs to address better upon discharging patients home from the hospital?**

**Closing**

These are all of the questions we have. Is there anything else you would like to tell us before we conclude? Thank you for your time and for participating in the discussion.
